# Use of Chemometrics for Correlating Carobs Nutritional Compositional Values with Geographic Origin

**DOI:** 10.3390/metabo10020062

**Published:** 2020-02-10

**Authors:** Rebecca Kokkinofta, Stelios Yiannopoulos, Marinos A. Stylianou, Agapios Agapiou

**Affiliations:** 1 State General Laboratory, P.O. Box 28648, 2081 Nicosia, Cyprus; 2 Department of Chemistry, University of Cyprus, P.O. Box 20537, 1678 Nicosia, Cyprus

**Keywords:** nutrients, food constituents, differential characteristics, multivariate data analysis

## Abstract

Carobs unique compositional and biological synthesis enables their characterization as functional foods. In the present study, 76 samples derived from fruit and seeds of carobs, with origin from the countries of the Mediterranean region (Cyprus, Greece, Italy, Spain, Turkey, Jordan and Palestine) were analyzed for their nutritional composition, in order to identify potential markers for their provenance and address the carobs’ authenticity issue. Moisture, ash, fat, proteins, sugars (fructose, glucose, sucrose), dietary fibers and minerals (Ca, K, Mg, Na, P, Cu, Fe, Mn, Zn) were estimated following official methods. Due to the large number of data (76 samples × 17 parameters × 7 countries), chemometric techniques were employed to process them and extract conclusions. The samples of different geographical origin were discriminated with 79% success in total. The carobs from Cyprus, Italy and Spain were correctly classified without error. The main discriminators were found to be the dietary fibers, the carbohydrates and Cu, Zn and Mn, which emphasize their specific nutritional added value to the product and the country of origin impact. The results suggest that the proposed analytical approach is a powerful tool that enables the discrimination of carobs based on their country of origin. This research contributes to authenticity of carobs, adding value to local products.

## 1. Introduction

The last decade, consumers appear to be more concerned and demanding in regard to their nutrition, as a result of the various food frauds and diseases (e.g., obesity, diabetes, heart attacks, etc.) associated with modern food [1]. As it was emphasized, the answer to food authentication, contamination and spoilage can be approached only through the continued improvement of the multidisciplinary approaches of analytical, computer and engineering sciences, as the respective issue remains always global and timely [2]. 

Driven by modern health trends (e.g., biological-, gluten- and caffeine-free products) and the high demand for natural hydrocolloids, there is an urgent request for authentic, biological and functional foods, following the basic aspects of the Mediterranean diet. *Ceratonia siliqua L.* is considered a multi-factory tree, serving both the needs for a high nutritional and biological product; therefore, it seems to fulfill the above needs [3]. Although it has been neglected the last decades and survived mainly as a food for animals, it dynamically returns to the global market as a potential functional and nutraceutical food-product, serving and satisfying the modern health and current nutritional demands. Carob flesh is rich in minerals, dietary fibers, sugars, polyphenols, D-pinitol and with low fat content, whereas the seed is abundant with locust bean gum (E 410), a natural hydrocolloid widely used in the food industry [3,4,5,6,7]. The chemical composition of carob flesh is presented in Table 1; however, these values are affected by various practices and factors (e.g., farming, genotyping, climate, soil, etc.). Environmentally speaking, the wide presence of carobs in the Mediterranean region can be attributed to combat the negative effects of climate change. The biological importance of carob leaves as a potential antifeedant was recently revealed [8]. The complexity of carob metabolome in combination with origin, ripening and roasting was studied using hyphenated state-of-the-art liquid and gas mass spectrometric systems [9]. The positive biological effects of carobs in the gastrointestinal tract, consumed either as water diluted food [10] or as an alcoholic drink [11], is still triggering the interest of researchers. Last but not least, is the carob’s unique sensorial characteristics, as both the fruit and the carob-products, are associated with a strong and persisting unique aroma [12].

Initially it was abundantly cultivated only in the eastern Mediterranean countries, but nowadays, due to human immigration and distribution, is grown far away from the Mediterranean region (e.g., in Africa, Australia, Asia, USA). Nevertheless, is still considered a characteristic indigenous tree, next to olives, almond and figs, enabling even the production of local beverages as carob liqueurs [21]. Cyprus, is considered among the top-10 carob producing countries, with a long history on carob production and processing. Thus, a wide array of traditional carob foods and products are developed, advantaging the unique nutritional properties of carob fruit [22]. 

Currently, in Europe, there is a trend towards documenting, supporting, labelling and highlighting the respective traditional products in order to protect, enhance and raise their market value. The geographical origin of foods is an issue of high concern and interest, as specific food labels are nowadays promoted; protected designation of origin (PDO), protected geographical indication (PGI), and traditional specialities guaranteed (TSG) [1]. Towards this, the various available extraction techniques (e.g., liquid-liquid-extraction (LLE), solid-phase-extraction (SPE), solid-phase micro- extraction (SPME), stir bar sorptive-extraction (SBSE), microwave-assisted extraction (MAE), as well as the quick, easy, cheap, effective, rugged and safe extraction (QuEChERS)), are associated by an array of modern analytical methods (e.g., mass spectrometry, spectroscopy, chromatographic and hyphenated, electronic sensors) and chemometric tools (e.g., exploratory analysis, classification/discriminant analyses, regression analyses/prediction models) for monitoring and protecting food adulteration and authentication [23]. In this context, FT-IR spectroscopy when combined with chemometrics, allowed the direct classification of carobs per country [24]. Similarly, the non-destructive headspace SPME gas chromatography mass spectrometry (HS-SPME/GC-MS) method was employed to discriminate the white rice originated from Korea and China based on the emitted volatiles [25]. Chemometrics were also applied to selected ion flow tube mass spectrometry (SIFT-MS) data, in order to correlate the complex aroma of roasted coffee from seven different countries [26]. The latter, was achieved despite the strong effects of cultivar, agriculture practices and local micro-climate (e.g., sun, water, soil, etc.) to the physical and physicochemical properties of the fruit. Lately, a number of metabolites (<1200 Da) were reviewed as potential authenticity and safety indicators for the food domain [27]. The increasing popularity of carobs in combination to their commercial added value, requires the protection of their authenticity towards their production from other countries. Therefore, new analytical tools need to be explored to enhance carobs economic sustainability. The present study attempts to enlighten the nutritional compositional value of carobs, in order to develop a useful tool to characterize and support their potential geographic origin certification. Therefore, the analysis of nutritional composition of fruit and seeds of carobs from the Mediterranean region were further processed with various chemometric tools in order to identify potential markers for their authenticity.

## 2. Results and Discussion 

The carob samples were obtained from seven Mediterranean countries (Cyprus, Greece, Italy, Spain, Turkey, Jordan and Palestine). The exploratory principal component analysis (PCA) was firstly applied on 17 nutritional values (moisture, ash, fat, proteins, fructose, glucose, sucrose, dietary fibers and Ca, K, Mg, Na, P, Cu, Fe, Mn, Zn), derived from 76 samples. The respective analytical data are presented in Table S1 (Supplementary Materials). The achieved scores were further employed to classify the samples into one pre-formed groups (based on their geographical origin or their type). This analysis gives an understanding of the relationship among the variables and shows which of these provide similar information to the proposed model and which offer unique information. The extracted model with two components calculated from the correlation matrix explained the 57.1% of the total variation, while the first five components explained more that 78.1% of the total variation (Table 2). Table 2 reports the cumulative percentage of the total variance provided by the first 10 principal components obtained from the whole data set.

**Figure 1:**
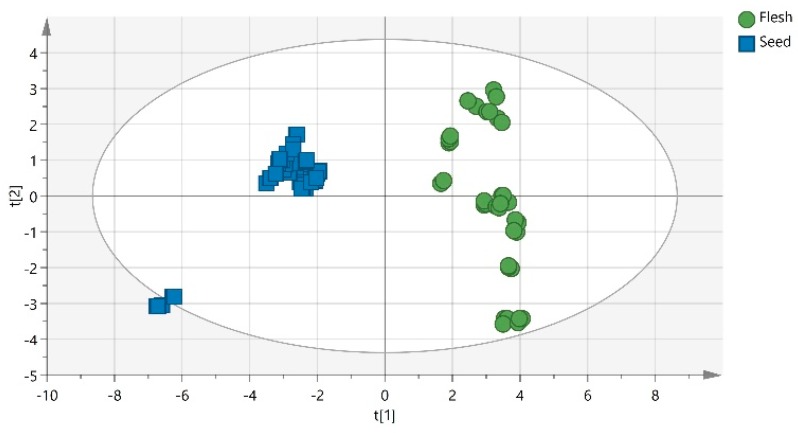
PCA scatter plot for 76 carob samples (R^2^X (cum) = 0.72, Q^2^ (cum) = 0.50).

PCA scatter plot (Figure 1) shows a clear separation of carob seeds from flesh samples based on the highest values of proteins, dietary fibers, fat, Ca, Zn, ash, Cu, Mn, P, Fe, Mg and the lower values of moisture, K, Na, sucrose, glucose, fructose. 

To proceed a step further, the existing information was used for a smaller number of carobs (only 54 samples, that of known varieties), in order to investigate the effect of botanical origin (cultivars) on their nutritional value (Figure S1). The results support that nutritional value varies more among carobs of different geographical origin than carobs of different botanical origin (variety). Of course, it should be noted that there were no samples of the same variety from different countries. Therefore, there is a direct relationship between the varieties and the country of origin. A similar conclusion can be drawn from Figure S2, where the contribution plots of the 12 varieties based on the PCA model (Figure S1) are presented. The parameters with a score higher than 1.0 are highlighted.

**Figure 2:**
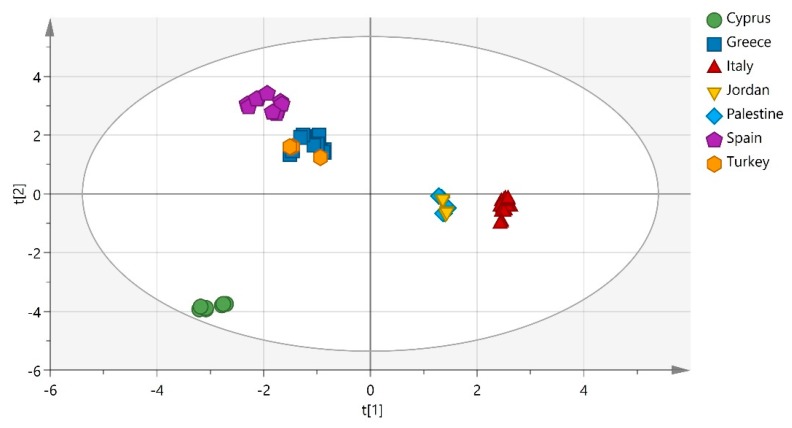
Partial least square-discriminant analysis model, according to origin (R^2^X (cum) = 0.70, Q^2^ (cum) = 0.91), discriminating Cypriot carobs from Italy and Spain.

**Figure 3:**
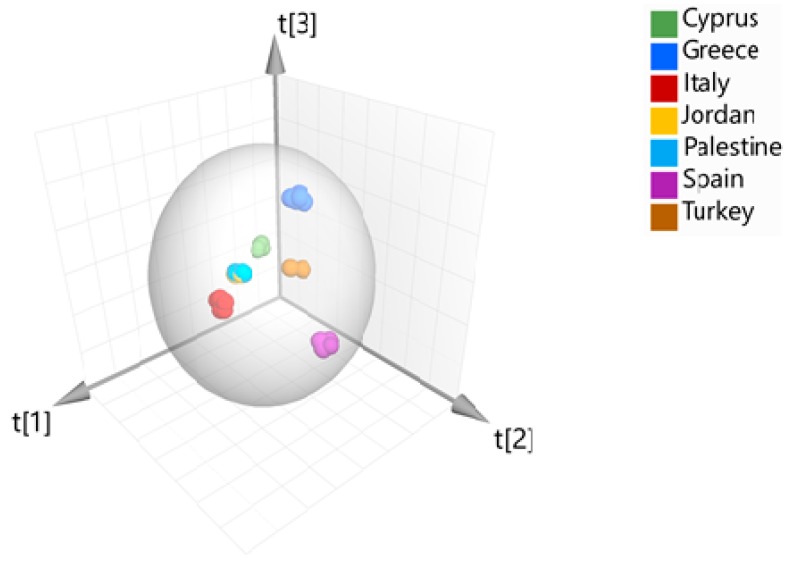
Orthogonal partial least square-discriminant analysis model (3D plot) (*N* = 76, R^2^X (cum) = 0.59, Q^2^ (cum) = 0.60), discriminating the groups of carobs from Cyprus, Italy, Spain, Greece and Turkey.

**Figure 4:**
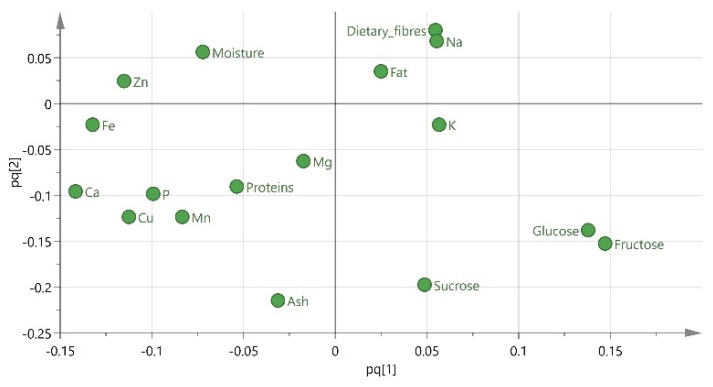
Loading score plot of the OPLS model.

Then, partial least square-discriminant analysis (PLS-DA) and orthogonal partial least square-discriminant analysis (OPLS-DA) were successfully applied to discriminate the samples according to their geographical origin, with OPLS-DA giving the best discrimination. The extracted models are presented in Figure 2 and Figure 3 and the parameters that characterize each group of samples are showed to the respective loading plot (Figure 4). The three groups of samples from Cyprus, Italy and Spain have been successfully discriminated. They consisted of 2 subgroups each, flesh and seeds, whereas for the samples from Greece and Turkey, this discrimination was not clear. Finally, the samples from Jordan and Palestine failed to distinguish correctly and half of the samples from each country appeared to be grouped with the samples from the other country. The main discriminators for Cypriot carobs were found to be Cu and Mn, while fructose and glucose for the Italian carobs and finally Zn for the Spanish carobs. This is quite interesting, as in the past, it was concluded that the minerals of Mg, Zn and Cu highly contributed to the authenticity of *“Zivania”,* a traditional Cypriot spirit [28]. The existence of high amounts of copper in Cyprus (*aes cyprium* = Cypriot metal) from the ancient years is well known and documented [29]. 

**Figure 5:**
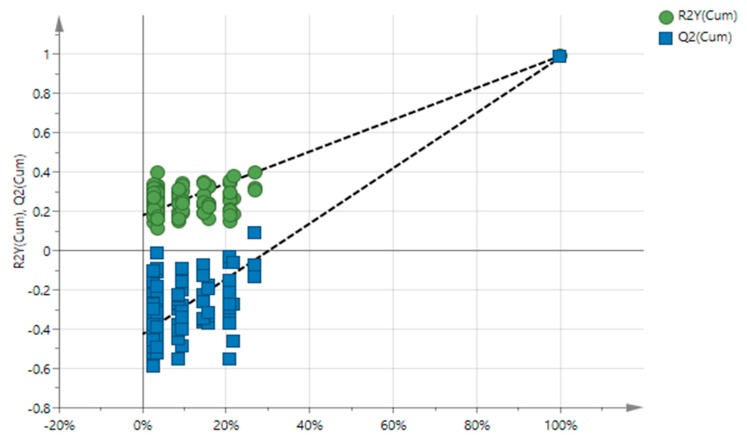
Permutation plot of the PLS model (R^2^ = 0.95%, Q^2^ = 0.98).

The permutation plot (Figure 5) assesses the risk that the obtained PLS is valid and predicts well for new observations; intercepts R^2^ = (0.0, 0.18) and Q^2^ = (0.0, −0.425). The model’s efficiency was defined by the goodness-of-fit R^2^ = 0.95%, whereas predictive ability by Q^2^ = 0.98. The model has been confirmed using cross validation-analysis of variance (CV-ANOVA), with a *p*-value < 4.35 × 10^−5^. The misclassification table presented in Table 3 was calculated for validation purposes. The samples of different geographical origins were discriminated with 78% success in total and the Fisher’s probability is highly satisfactory (3.7 × 10^−7^ < 0.05).

Chemometrics is a powerful analytical tool widely employed in the field of food science (e.g., analysis/authenticity, microbiology, processing, etc.), where big and complex experimental data are produced [30]. Nutritional parameters are relatively cheap and routine analytical measurements are carried out daily from the food laboratories over the world, for revealing the respective food composition and quality. What is unnoticed however, is that these characteristic nutritional values can play an alternative novel role in the food domain. Actually, when combined with appropriate chemometric tools, they obtain the potential to become an interesting dynamic tool, offering extra information for food authenticity and origin. The various analytical methods (e.g., spectroscopic, spectrometric, chromatographic, etc.) employed in food analysis are costly and end up with a specific result. Therefore, in most cases, extra analysis is requested with supplementary instruments. The more information gathered for a food component, the more solid the result can become; as multiple data of compositional analysis are gathered daily in food chemistry laboratories, chemometrics can reveal the respective hidden correlations.

## 3. Conclusions

Since the era of functional and nutraceutical foods is ahead, carob nutrient synthesis is examined next to chemometrics, for classifying carobs based on their geographical origin. The huge amount of gathered data and samples were further processed in order to identify possible hidden correlations. PCA enabled to distinguish carob seeds from flesh samples, based on the highest values of proteins, dietary fibres, fat, Ca, Zn, ash, Cu, Mn, P, Fe, Mg and the lower values of moisture, K, Na, sucrose, glucose, fructose. The samples of different geographical origin were discriminated by PLS with 78% success in total. There was 100% correct classification in 4 of the 7 groups: carobs from Cyprus, Greece, Italy and Spain, while Jordanian and Palestinian carobs were presented together in one group. Carobs from Turkey were classified as Greek, but it should be noted that the number of samples of this origin was very small, only 4. The main discriminators for Cypriot carobs were found to be Cu and Mn, while fructose and glucose for the Italian carobs and finally Zn for the Spanish carobs. The combined employment of chemometrics and chemical composition mapping enable to distinguish carobs based on their country of origin. The potential of the developed methodology to be further applied at the broader food science domain remains to be explored. 

## 4. Materials and Methods 

### 4.1. Samples

In total, 76 carob samples (seeds and flesh) were obtained from 7 countries of the broader Mediterranean area (Cyprus, Greece, Italy, Spain, Turkey, Jordan and Palestine). All the samples were authentic with valid information regarding their origin and cultivar. They were analyzed for their nutritional composition, in order to identify markers for their authenticity. A variety of cultivars were used from each country (Table 4). 

### 4.2. Nutritional Composition Analysis 

An investigation of the basic nutritional parameters of carobs was carried out. Therefore, the analyses were performed in an accredited laboratory employing official or accredited/validated analytical methods. Seventeen basic nutritional parameters were examined: moisture, ash, fat, proteins, sugars (fructose, glucose, sucrose), dietary fibers and minerals (Ca, K, Mg, Na, P, Cu, Fe, Mn, Zn). For the respective determinations, the following experimental procedures were followed; however, more details can be also found at [22]. 

Moisture content was carried out by drying the samples in an oven (Gallenkamp) at 130 ± 2 °C according to AOAC 925.10 [31] and AACC 44-15A [32] official methods of analysis. For moisture content analysis, it was checked whether the gravimetric difference between the samples was less than the reproducibility of the method, while the process was repeated three times.

Ash content was estimated by igniting the samples in a muffle furnace (Carbolite furnace) at 550 °C for 5 h based on the 14-098 method [33].

Proteins content was determined according to AOAC methods 991.20-2011 [34], 920.87-2010 [35] and ISO 937-1978 [36] using Buchi Autokjeldahl Unit K-370. Wheat flour—certified reference material FAPAS T2410, regulatory standard solutions for pH = 4 and pH = 7, secondary reference material (ammonium sulfate) and interlaboratory tests were followed. The conversion factor of 6.25 was finally applied for the conversion of Total-N to crude protein. 

Carbohydrates (fructose, glucose, sucrose) determination was based on AOAC 977.20 [37] and measured through HPLC (Alliance, Waters 2695; Waters 2414 Refractive Index Detector; Waters Spherisorb NH_2_ (4.6 mm × 250 mm × 10 μm) analytical column, Waters, Milford, MA, USA). An isocratic mobile phase was followed consisting of CH_3_CN:H_2_O (87:13 *v/v*), with flow rate of 1 ml/min at 35 °C. High purity internal standard solutions of Sucrose (Bioxtra ≥99.5%), D(-)Fructose (≥99.0%), D(+)Glucose (99%) from Sigma-Aldrich were used for the qualitative and quantitative determinations. Towards this, different standard solutions (g/100 mL) were prepared in order to develop the respective calibration curves.

Fat content was determined in two steps: (a) acid hydrolysis and (b) solvent extraction using petroleum ether (Sigma-Aldrich) as solvent in the Soxhlet extraction system (BUCHI extraction Unit E-816 SOX) for 1 h, according to the official AOAC methods 991.36 [38] and 963.15 [39].

Total dietary fibers were measured through the enzymatic gravimetric method as described to the official AOAC standard procedure 985.269 [40] and AACC 32-05.01 [41]. For interlaboratory testing, enzymes a-amylase, protease and amyloglucosidase by Megazyme (Dietary Fiber Kit) were employed along with wheat flour—certified reference material FAPAS T2438 and FAPAS T2442. The difference between the samples was checked to be less than the reproducibility of the method.

Minerals composition was determined with Inductively Coupled Plasma Atomic Emission Spectroscopy ICP/OES (Thermo Scientific Icap 6000 SERIES) based on AOAC 985.01 [42] and AOAC 984.27 [43]. About 0.5 g dried and ground sample was placed into a reference polypropylene segment cup and 7 mL pure HNO_3_ (Carlo Erba) and 1 mL of H_2_O_2_ (Merck) were added. The sample was incinerated in an ETHOS 1 Microwave digestion system at 200 °C and the solution was diluted to a certain volume (50 mL) with distilled water. Certified internal standards were used to determine the minerals’ concentrations (Ca, K, Mg, P, Cu, Fe, Mn, Zn, Na).

During the measurements, internal and external quality control was performed to ensure the accuracy and reliability of the results. The individual result for each carob sample was expressed with the method uncertainty. All the foreign carob samples were analyzed in triplicate, except Cyprus samples that were duplicated measured. 

### 4.3. Multivariate Data Analysis (MDA)

Each sample was considered as an assembly of seventeen variables represented by the nutritional data. The MDA was carried out using SIMCA statistical software package (version 15.02, Umetrics, Umeå, Sweden) [44]. The pattern recognition tools implemented in [45] were applied. 

PCA is an unsupervised technique that identifies correlations between variables in the data, highlighting their similarities and differences and reduces the dimensions of the data while retaining the significant ones [46,47,48]. Using as input data, the new principal components that were mean-centered with unit variate (UV) scaling, the PCA model was extracted at a confidence level of 95%. PCA provides classification models through the soft independent modeling of class analogy (SIMCA). 

PLS-DA is a supervised method that can be used for predictive and descriptive modeling, as well as for discriminative variable selection. The systematic variation in X is separated into two parts, the linearly related to Y (predictive information) and the unrelated to Y, building a new classification model. The most discriminant variables are highlighted through the resulting loading and contribution diagrams.

The extracted PLS-DA models at a confidence level of 95% were UV scaled and log transformed. The efficiency of the models was first evaluated by calculation of the goodness-of-fit R^2^ and the predictive ability of the model Q^2^. The variation R^2^ (0 ≤ R^2^ ≤ 1) explains the quality of the models (PCA, PLA), how well the data of the training set is mathematically reproduced, while the cumulative Q^2^ (0 ≤ Q^2^ ≤ 1) represents the fraction of the variation of Y that can be predicted. The cross validation-analysis of variance (CV-ANOVA), expressed as a *p*-value <0.05, was further applied to validate the models; the produced misclassification table highlighted the model’s performance (classification error).

The OPLS-DA improves the classification model quality by separating the systematic variation in X into two parts; the predictive information linearly related to Y and the uncorrelated information unrelated to Y (orthogonal information). This results to improve diagnostics and interpreted visualization. The advantage of OPLS-DA is that only a single component is the predictor for a particular class, while the rest of the components describe the variation orthogonal to this first predictive component.

The extracted OPLS-DA models were UV scaled and log transformed. Loading plot was extracted to highlight the most discrimination variables and the variation R^2^ and the cumulative Q^2^ were calculated by the internal cross validation method of SIMCA software. The OPLS models have been validated using CV-ANOVA, with a *p*-value < 0.05. The application of OPLS-DA, improved the interpretability of the PLS models, not their predictivity.

## Figures and Tables

**Table 1:** Composition of carob (*Ceratonia siliqua L.*) flesh based on literature data.

Chemical Composition (% w/w)								
Sucrose	Fructose	Glucose	Dietary Fiber	Ash	Protein	Fat	Moisture	Source
35–45	6–7	2–4	Up to 40	2–3	2–7	0.5–1	-	[3]
40.7–54.7 (sugars) ^1^			7.6–38.0	2.0–3.4	2.0–7.6	0.4–1.3	6–11	[13]
75.9 (carbohydrates) ^2^			7.3	3.2	6.3	2.0	5.3	[14]
48.3 (total sugars) ^3^			9.7	3.3	4.7	0.2	6.0	[15]
35	7	5	-	2	2	0.4	6	[16]
79.8 (carbohydrates) ^2^			9.1	3	6.8	1.2	-	[17]
31.5–50.1 (total sugars) ^3^			-	2.4–3.9	3.1–4.5	0.5–0.8	12.2–13.5	[18]
33.7–45.1	1.8–5.2	1.8–4.9	29.9–36.1	2.1–2.4	3.1–4.4	0.4–0.9	8.2–9.6	[19]
32.6–45.4	6.9–7.4	2.0–2.3	6.8–7.1	2.7–3.2	2.6–2.8	1–1.3	9.7–10.7	[20]

1, 2, 3 = as indicated in the respective manuscripts.

**Table 2:** Principal component analysis: cumulative proportion of total variation (%) calculated from correlation matrix by Nipals.

Component	1	2	3	4	5	6	7	8	9	10
**Cumulative**	45.5	57.1	65.6	72.8	78.1	82.4	86.6	90.7	92.8	95.0

**Table 3:** Misclassification table for the PLS model.

Class	No of Samples	Correct (%)	1	2	3	4	5	6	7	No Class (YPred ≤ 0)
**1 (Cyprus)**	12	100	12	0	0	0	0	0	0	0
**2 (Greece)**	12	100	0	12	0	0	0	0	0	0
**3 (Italy)**	24	100	0	0	24	0	0	0	0	0
**4 (Spain)**	12	100	0	0	0	12	0	0	0	0
**5 (Palestine)**	6	0	0	0	6	0	0	0	0	0
**6 (Jordan)**	6	0	0	0	6	0	0	0	0	0
**7 (Turkey)**	4	0	0	2	0	2	0	0	0	0
**No class**	0		0	0	0	0	0	0	0	0
**Total**	76	78.95	12	14	36	14	0	0	0	0
**Fisher’s probability 3.7 × 10^−7^**										

**Table 4:** Country of origin and cultivars of carobs.

Country	Cultivars	Sample Type
Cyprus	*Tylliria, Koumpota, Kountourka*	Flesh and seed
Greece	*Imera, Imera, Unknown*	Flesh and seed
Italy	*Raexmosa, Giubiliana, Saccarata, Unknown*	Flesh and seed
Spain	*Negra, Rojal, Metalafera*	Flesh and seed
Turkey	*Fleshy*	Flesh and seed
Jordan	*Unknown*	Flesh and seed
Palestine	*Unknown*	Flesh and seed

## Supplementary Materials

The following are available online at https://www.mdpi.com/2218-1989/10/2/62/s1: Figure S1: PCA scatter plot of 54 carob samples, according to their variety (C: Cyprus, G: Greece, I: Italy, S: Spain, T: Turkey). Figure S2: Contribution plots of the 12 varieties based on the PCA model (Figure S1). Table S1: Results obtained from the analyses of carobs (flesh and seeds).
