# Supplementary material for: Use of Chemometrics for Correlating Carobs Nutritional Compositional Values with Geographic Origin
Source: Metabolites. 2020 Feb 10;10(2):62. doi: 10.3390/metabo10020062 (PMC7074360; doi:10.3390/metabo10020062)
Supplement: Supplementary file 1 [file metabolites-10-00062-s001.pdf]

Supplementary Material

Use of chemometrics for correlating carobs nutritional compositional values with geographic origin

Rebecca Kokkinofta [1,*], Stelios Yiannopoulos [1], Marinos A. Stylianou [2], Agapios Agapiou [2,*]

[1] State General Laboratory, P.O.Box 28648, Nicosia, Cyprus
[2] University of Cyprus, Department of Chemistry, P.O. Box 20537, 1678, Nicosia, Cyprus
* Correspondence: sglsnif@cytanet.com.cy (R.K.); agapiou.agapios@ucy.ac.cy (A.A.)

Received: date; Accepted: date; Published: date

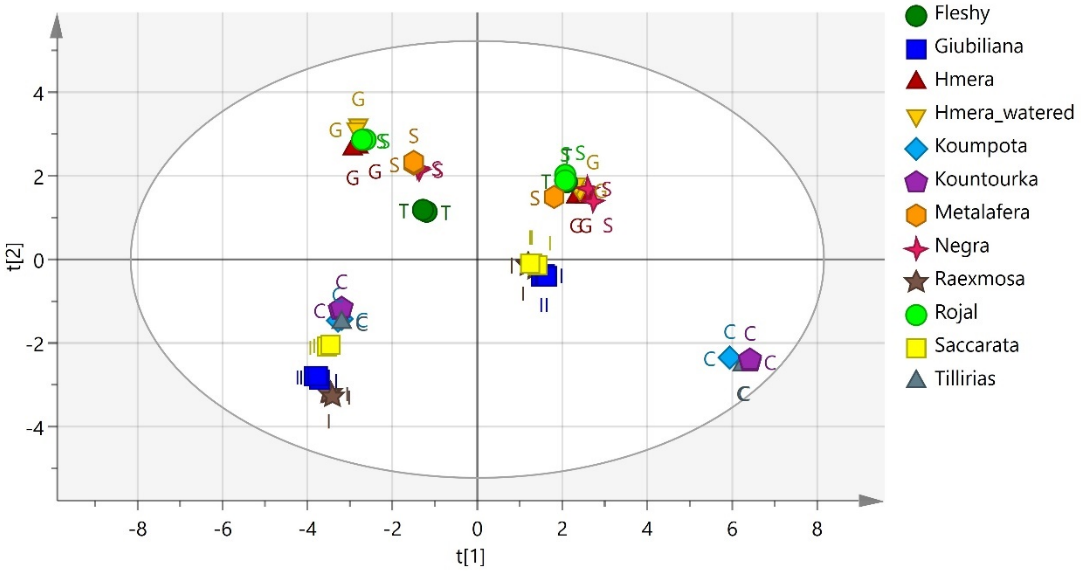

Figure S1: PCA scatter plot of 54 carob samples, according to their variety (C: Cyprus, G: Greece, I: Italy, S: Spain, T: Turkey).

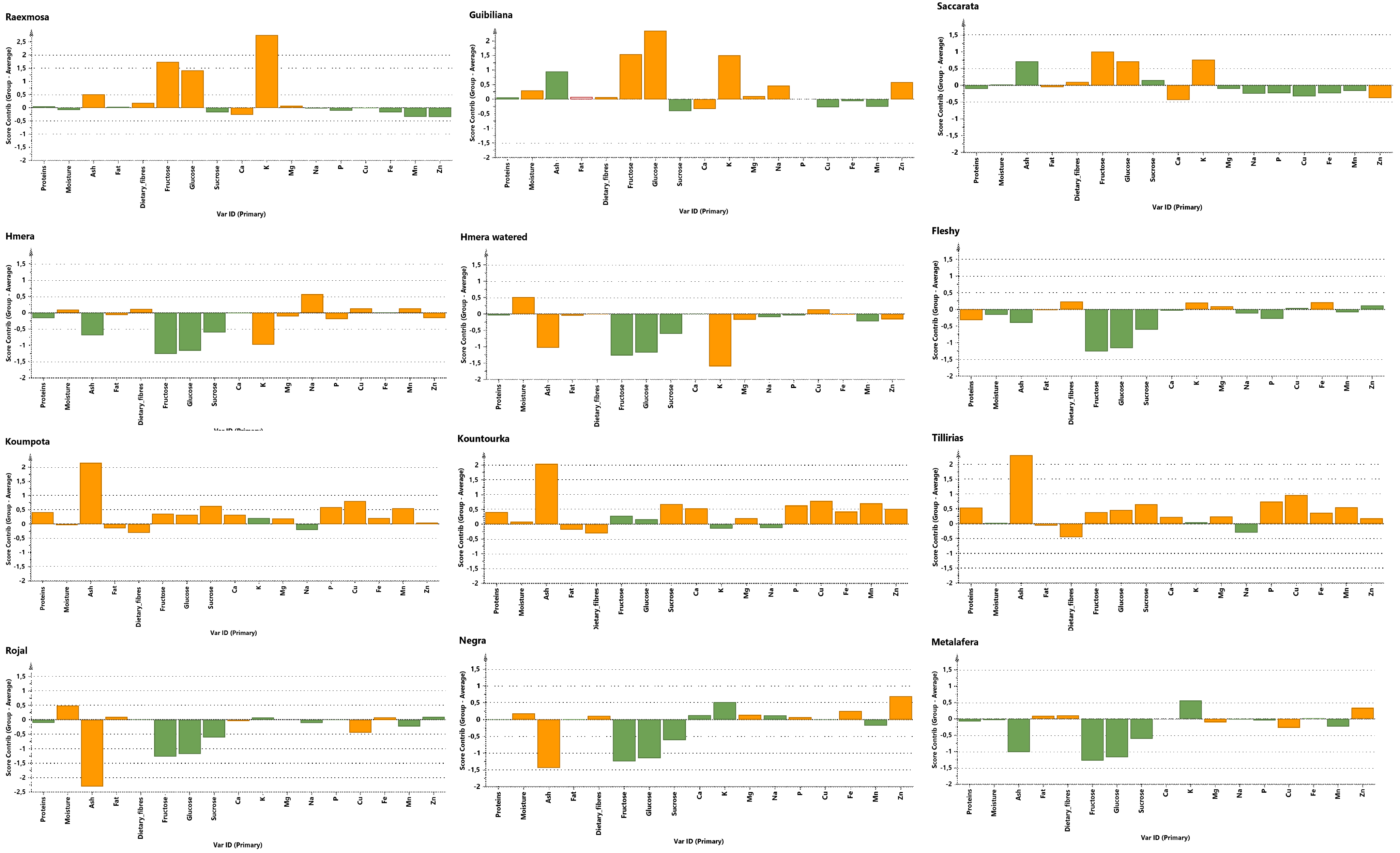

Figure S2: Contribution plots of the 12 varieties based on the PCA model (Fig. S1).

Table S1: Results obtained from the analysis of carobs (flesh and seeds).

			Proteins	Moisture	Ash	Fat	Dietary fibres	Fructose	Glucose	Sucrose	Ca	K	Mg	Na	P	Cu	Fe	Mn	Zn
**Country**	**Type**	**Variety**	(% w/w)								Mineral content (mg/100 g)								
**Palestine**	Seed	Uknown	13.73	9.90	3.12	1.92	64.12	0.00	0.00	1.02	364.00	868.00	119.00	<1	230.00	0.53	3.03	2.66	2.14
**Palestine**	Seed	Uknown	13.39	10.09	3.06	1.90	63.01	0.00	0.00	0.65	385.00	900.00	126.00	<1	240.00	0.52	3.01	2.69	2.74
**Palestine**	Seed	Uknown	13.54	9.89	3.00	1.84	62.53	0.00	0.00	0.86	375.00	887.00	123.00	<1	238.00	0.52	3.12	2.73	2.64
**Palestine**	Flesh	Uknown	3.70	11.46	2.75	0.22	21.34	10.27	4.15	40.18	451.00	741.00	83.60	6.12	57.50	0.20	0.85	0.39	0.55
**Palestine**	Flesh	Uknown	3.33	11.20	2.67	0.21	21.92	10.51	4.07	41.91	467.00	740.00	78.70	3.32	56.90	0.18	0.83	0.40	0.54
**Palestine**	Flesh	Uknown	3.36	11.23	2.71	0.18	21.24	9.91	4.15	41.84	453.00	734.00	78.10	3.22	56.10	0.18	0.78	0.39	0.56
**Jordan**	Seed	Uknown	16.78	9.58	3.22	2.25	63.46	0.00	0.00	2.04	411.00	883.00	129.00	<1	228.00	0.51	2.79	2.58	2.31
**Jordan**	Seed	Uknown	17.10	9.43	2.95	2.29	62.47	0.00	0.00	0.00	455.00	910.00	136.00	<1	242.00	0.53	2.93	2.69	2.26
**Jordan**	Seed	Uknown	16.75	9.84	3.00	2.26	62.32	0.00	0.00	0.00	380.00	859.00	124.00	<1	228.00	0.51	2.71	2.42	2.10
**Jordan**	Flesh	Uknown	3.53	10.40	2.75	0.25	24.91	10.76	4.60	38.89	443.00	773.00	80.90	3.36	58.30	0.19	0.99	0.42	0.74
**Jordan**	Flesh	Uknown	3.22	10.54	2.68	0.28	24.76	10.76	4.27	39.98	447.00	795.00	83.80	4.65	59.10	0.20	1.02	0.43	0.76
**Jordan**	Flesh	Uknown	3.15	10.30	2.61	0.24	24.35	10.44	4.50	40.57	419.00	786.00	80.30	3.63	56.20	0.19	0.94	0.42	0.70
**Italy**	Seed	Uknown	10.96	9.95	2.84	1.69	68.35	0.00	0.00	0.00	417.00	785.00	119.00	1.81	207.00	0.65	2.91	2.36	1.75
**Italy**	Seed	Uknown	10.76	9.90	2.96	1.63	69.68	0.00	0.00	0.00	408.00	768.00	118.00	2.81	207.00	0.65	3.05	2.40	1.85
**Italy**	Seed	Uknown	11.13	9.86	3.09	1.69	68.96	0.00	0.00	0.00	439.00	818.00	129.00	1.40	216.00	0.67	3.27	2.53	1.95
**Italy**	Flesh	Uknown	5.51	9.15	2.76	0.43	28.83	6.42	1.60	39.04	304.00	815.00	50.70	3.63	61.00	0.30	1.30	0.57	0.56
**Italy**	Flesh	Uknown	5.33	9.35	2.62	0.37	28.65	6.40	1.78	39.43	328.00	808.00	56.70	3.79	59.70	0.30	1.32	0.62	0.60
**Italy**	Flesh	Uknown	5.43	9.06	2.52	0.35	29.15	5.71	1.67	39.79	334.00	815.00	56.50	3.90	61.60	0.29	1.31	0.60	0.58
**Italy**	Seed	Raexmosa	14.89	10.69	2.76	1.78	65.13	0.00	0.00	1.53	342.00	878.00	150.00	<1	214.00	0.55	2.93	1.27	1.68
**Italy**	Seed	Raexmosa	16.21	10.74	2.77	1.67	67.49	0.00	0.00	1.36	377.00	917.00	158.00	<1	228.00	0.57	2.87	1.29	1.74
**Italy**	Seed	Raexmosa	15.52	10.64	2.87	1.70	64.32	0.00	0.00	1.48	370.00	823.00	145.00	<1	201.00	0.53	2.63	1.23	1.70
**Italy**	Flesh	Raexmosa	5.91	11.46	3.54	0.58	35.03	17.47	6.76	13.14	121.00	1479.00	67.10	4.54	97.80	0.40	1.43	0.35	0.83
**Italy**	Flesh	Raexmosa	5.82	11.37	3.59	0.47	35.62	17.73	7.14	13.24	123.00	1440.00	65.40	4.34	97.70	0.40	1.32	0.36	0.81

**Italy**	Flesh	Raexmosa	5.37	11.47	3.89	0.56	35.08	17.91	6.99	13.07	125.00	1441.00	65.10	3.99	96.40	0.39	1.32	0.36	0.96
**Italy**	Seed	Giubiliana	15.26	11.07	3.14	1.88	66.43	0.00	0.00	1.08	303.00	938.00	166.00	<1	264.00	0.53	3.29	1.50	1.80
**Italy**	Seed	Giubiliana	16.23	11.10	3.18	1.79	64.26	0.00	0.00	0.00	291.00	898.00	159.00	<1	257.00	0.53	3.88	1.47	2.18
**Italy**	Seed	Giubiliana	16.31	11.17	3.05	1.90	63.90	0.00	0.00	0.00	313.00	943.00	165.00	<1	261.00	0.55	3.49	1.59	1.97
**Italy**	Flesh	Giubiliana	5.62	14.50	3.66	0.55	30.59	16.91	9.46	6.67	120.00	1202.00	63.50	8.33	85.30	0.24	1.38	0.38	4.22
**Italy**	Flesh	Giubiliana	5.40	14.52	3.63	0.47	29.85	16.12	9.51	6.96	113.00	1176.00	58.20	8.75	86.90	0.24	1.30	0.38	4.12
**Italy**	Flesh	Giubiliana	5.43	14.34	3.65	0.58	30.48	16.52	9.33	6.61	118.00	1168.00	57.20	8.96	85.80	0.24	1.35	0.39	4.31
**Italy**	Seed	Saccarata	14.16	10.58	3.11	1.75	65.03	0.00	0.00	1.66	313.00	855.00	127.00	<1	221.00	0.53	3.00	1.92	1.97
**Italy**	Seed	Saccarata	14.33	10.67	3.06	1.79	63.84	0.00	0.00	0.93	341.00	878.00	135.00	<1	225.00	0.53	3.27	2.00	1.87
**Italy**	Seed	Saccarata	14.63	10.53	3.04	1.70	65.50	0.00	0.00	1.35	310.00	850.00	127.00	<1	216.00	0.54	2.84	1.89	1.84
**Italy**	Flesh	Saccarata	4.45	12.43	3.55	0.28	30.23	13.67	5.14	23.06	165.00	1135.00	59.80	3.19	63.80	0.23	1.35	0.42	0.62
**Italy**	Flesh	Saccarata	4.25	12.29	3.58	0.30	32.33	13.37	5.23	23.29	159.00	1122.00	61.10	2.61	63.50	0.24	1.17	0.39	0.72
**Italy**	Flesh	Saccarata	4.28	12.47	3.55	0.31	31.06	13.63	5.11	23.54	169.00	1119.00	61.90	2.33	63.50	0.24	1.15	0.42	0.67
**Greece**	Seed	Hmera	12.82	8.80	3.14	1.61	69.08	0.00	0.00	0.00	408	923	116.9	2.60	233	0.79	4.22	2.75	2.73
**Greece**	Seed	Hmera	13.03	8.96	3.22	1.64	68.15	0.00	0.00	0.00	403	901	144.66	1.60	229.1	0.75	4.08	2.83	2.69
**Greece**	Flesh	Hmera	3.70	14.85	2.45	0.26	29.56	0.138	0.187	0.746	264	774	45.87	8.80	46.2	0.27	1.36	0.57	0.55
**Greece**	Flesh	Hmera	3.65	15.13	2.56	0.28	28.96	0.131	0.201	0.747	256	763	45.55	10.10	45.5	0.29	1.29	0.56	0.55
**Greece**	Seed	Hmera_ watered	14.71	9.03	3.23	1.64	67.77	0.00	0.00	0.00	460	884	115.07	1.80	253	0.81	4.71	1.58	2.60
**Greece**	Seed	Hmera_ watered	15.52	8.94	3.07	1.83	65.37	0.00	0.00	0.00	446	857	111.6	1.50	245.6	0.81	3.97	1.55	2.54
**Greece**	Flesh	Hmera_ watered	4.29	19.18	2.32	0.17	23.73	0.12	0.156	0.618	296	679	48.82	2.20	69.9	0.24	0.81	0.26	0.64
**Greece**	Flesh	Hmera_ watered	4.11	19.16	2.31	0.26	25.22	0.107	0.158	0.621	264	732	42.91	3.50	93.8	0.26	0.92	0.33	0.71
**Greece**	Seed	Uknown	19.39	7.49	3.7	1.86	65.96	0.00	0.00	0.00	425	959	120.19	2.40	290.3	0.71	3.99	2.65	2.29
**Greece**	Seed	Uknown	17.07	7.76	3.69	1.75	65.71	0.00	0.00	0.00	423	947	116.51	1.10	280	0.70	3.99	2.64	2.27
**Greece**	Flesh	Uknown	4.36	12.49	2.28	0.39	27.48	0.072	0.147	0.746	251	743	42.44	3.30	84.1	0.25	1.04	0.45	0.65
**Greece**	Flesh	Uknown	4.16	12.52	2.26	0.47	27.70	0.07	0.148	0.76	267	697	48.57	2.20	70.7	0.28	1.20	0.46	0.77
**Turkey**	Seed	Fleshy	12.08	10.14	2.51	1.95	67.48	0.00	0.00	0.00	332.00	757.00	155.00	1.57	193.00	0.72	4.75	1.94	1.57
**Turkey**	Seed	Fleshy	10.83	10.01	2.58	1.85	68.14	0.00	0.00	0.00	348.00	752.00	159.00	1.39	192.00	0.68	4.77	1.96	1.39

**Turkey**	Flesh	Fleshy	2.29	11.22	3.4	0.15	38.73	0.064	0.16	0.62	321	1138	65.4	2.80	59.5	0.29	2.34	0.52	2.80
**Turkey**	Flesh	Fleshy	2.37	11.30	3.25	0.2	32.87	0.064	0.16	0.62	328	1114	65.2	2.97	59.1	0.27	2.37	0.53	2.97
**Cyprus**	Flesh	Koumpota	4.59	14.44	2.65	0.22	26.24	9.51	3.89	39.94	215	921	50	2.50	65	0.26	0.65	0.38	0.68
**Cyprus**	Flesh	Koumpota	4.55	14.50	2.59	0.21	-	9.67	4.29	39.88	215	921	50	2.50	65	0.26	0.65	0.38	0.68
**Cyprus**	Flesh	Kountourka	4.58	14.81	2.46	0.2	25.56	9.35	3.86	41.51	295	870	44	3.20	57	0.20	0.63	0.44	0.74
**Cyprus**	Flesh	Kountourka	4.51	14.85	2.51	0.22	-	8.92	3.44	40.96	295	870	44	3.20	57	0.20	0.63	0.44	0.74
**Cyprus**	Flesh	Tillirias	4.57	13.65	2.45	0.23	24.28	9.8	4.43	40.55	204	919	47	1.80	60	0.28	0.66	0.36	0.79
**Cyprus**	Flesh	Tillirias	4.63	13.59	2.46	0.23	23.77	9.73	4.47	40.67	204	919	47	1.80	60	0.28	0.66	0.36	0.79
**Cyprus**	Seed	Tillirias	25.04	9.46	5.25	1.77	46.33	0.00	0.00	0.00	641.1	935.4	194.5	<LOD (<1.3)	507.9	1.28	6.91	4.18	3.78
**Cyprus**	Seed	Tillirias	24.76	9.46	5.25	1.77	45.35	0.00	0.00	0.00	641.1	935.4	194.5	<LOD (<1.3)	507.9	1.28	6.91	4.18	3.78
**Cyprus**	Seed	Koumpota	23.16	8.08	4.97	1.55	50.39	0.00	0.00	0.00	708.4	961.4	185.2	<LOD (<1.3)	462.6	1.21	6.03	4.22	3.42
**Cyprus**	Seed	Koumpota	22.87	8.08	4.97	1.55	50.05	0.00	0.00	0.00	708.4	961.4	185.2	<LOD (<1.3)	462.6	1.21	6.03	4.22	3.42
**Cyprus**	Seed	Kountourka	22.48	8.69	5.05	1.53	51.43	0.00	0.00	0.00	713.3	949.9	189	<LOD (<1.3)	471.4	1.24	7.08	4.50	5.05
**Cyprus**	Seed	Kountourka	22.20	8.69	5.05	1.53	51.34	0.00	0.00	0.00	713.3	949.9	189	<LOD (<1.3)	471.4	1.24	7.08	4.50	5.05
**Spain**	Seed	Rojal	14.79	11.06	2.47	2.16	61.41	0.00	0.00	0.00	369.00	937.00	167.00	2.02	284.00	0.50	5.02	1.56	2.02
**Spain**	Seed	Rojal	13.40	11.66	2.84	2.16	62.80	0.00	0.00	0.00	375.00	953.00	169.00	1.77	286.00	0.50	5.16	1.59	1.77
**Spain**	Flesh	Rojal	3.79	16.35	1.89	0.4	29.67	0.067	0.144	0.609	210	920	37.27	2.62	58.30	0.14	1.32	0.28	2.62
**Spain**	Flesh	Rojal	3.74	16.74	2.06	0.4	30.13	0.064	0.146	0.611	169	907	33.61	2.25	58.80	0.13	1.40	0.26	2.25
**Spain**	Seed	Negra	16.10	10.37	2.25	1.65	64.37	0.00	0.00	0.00	468.00	956.00	173.00	3.69	303.00	0.70	4.62	1.78	3.69
**Spain**	Seed	Negra	15.98	9.81	2.86	1.71	59.16	0.00	0.00	0.00	468.00	956.00	173.00	3.69	303.00	0.70	4.62	1.78	3.69
**Spain**	Flesh	Negra	4.12	14.20	2.64	0.52	35.44	0.082	0.164	0.546	340	980	56.1	2.92	66.60	0.26	2.64	0.39	2.92
**Spain**	Flesh	Negra	4.10	14.62	2.6	0.52	35.14	0.079	0.167	0.546	340	980	56.1	2.92	66.60	0.26	2.64	0.39	2.92
**Spain**	Seed	Metalafera	14.79	9.84	2.86	2.17	24.92	0.00	0.00	0.00	423.00	928.00	144.00	1.80	276.00	0.61	4.15	1.50	1.80
**Spain**	Seed	Metalafera	14.70	9.95	2.84	2.21	24.90	0.00	0.00	0.00	423.00	928.00	144.00	1.80	276.00	0.61	4.15	1.50	1.80
**Spain**	Flesh	Metalafera	3.82	12.67	2.67	0.33	71.70	0.074	0.167	0.632	277	1012	34.6	3.48	54.70	0.15	1.44	0.34	3.48
**Spain**	Flesh	Metalafera	3.83	12.60	2.58	0.3	72.61	0.073	0.165	0.634	277	1012	34.6	3.48	54.70	0.15	1.44	0.34	3.48
